# An Efficient Transformation System for Fast Production of *VcCHS* Transgenic Blueberry Callus and Its Expressional Analysis

**DOI:** 10.3390/plants12162905

**Published:** 2023-08-09

**Authors:** Xuejing Qin, Jing Hu, Guohui Xu, Huifang Song, Lingyun Zhang, Yibo Cao

**Affiliations:** 1 State Key Laboratory of Efficient Production of Forest Resources, Key Laboratory of Forest Silviculture and Conservation of the Ministry of Education, The College of Forestry, Beijing Forestry University, Beijing 100083, China; krystal529142879@163.com (X.Q.); 18222617257@163.com (J.H.); songhuifang1010@163.com (H.S.); 2 College of Life and Health, Dalian University, Dalian 116000, China; xugh520@163.com

**Keywords:** callus induction, callus proliferation, *Agrobacterium* tumefaciens-mediated transformation, highbush blueberry

## Abstract

The *Agrobacterium* tumefaciens-mediated transformation for blueberries remains less efficient than is desirable. A new leaf callus regeneration and genetic transformation system was investigated in blueberries in this study. The leaf explants of *cv.* ‘Legacy’ and ‘Northland’ were used to establish the stable callus induction system when placed on the woody plant medium (WPM) supplemented with 1.0 mg·L^−1^ 2, 4-D, 0.4 mg·L^−1^ 6-BA for 30 d; then, the callus was sub-cultured in the proliferation medium supplemented with 1.5 mg·L^−1^ 2, 4-D, 0.4 mg·L^−1^ 6-BA in the darkness at 25 °C every 30 days. The co-cultivation of callus with *A. tumefaciens* was operated on WPM plus 100 μM acetosyringone for 4 days; then, the transferred callus was grown in WPM supplemented with 1.5 mg·L^−1^ 2,4-D, 0.4 mg·L^−1^ 6-BA, 50 mg·L^−1^ hygromycin, and 200 mg·L^−1^ cefotaxime. The *VcCHS* transgenic blueberry callus with both GFP signal and Hyg resistance was obtained from the transformed callus of *cv.* ‘Northland’. The rate of GFP signal detected in the transformed callus was as high as 49.02%, which was consistent with the PCR assay. Collectively, this study provides a highly efficient genetic transformation system in blueberry callus and a powerful approach for the molecular breeding of blueberries.

## 1. Introduction

Highbush blueberry (*Vaccinium corymbosum* L.), an important economically woody plant species known for its fruits, contains high amounts of antioxidants, anthocyanins, and various nutrients that have significant health benefits for the human body, which has driven industrial development around the world [1,2,3,4,5].

The process of breeding blueberries remains limited, due to high heterozygosity and the difficulty of regaining special traits from parent plants, as traditional methods are time-consuming and inefficient, although blueberry genetic improvement by genetic transformation methods is suitable [6,7,8,9]. The *Agrobacterium* tumefaciens-mediated transformation method has been extensively used in plant transformation [10]. It provides a powerful approach to increase breeding efficiency and improves the economic traits of blueberries, including fruit quality, yields, nutrient content, stress resistance, and the flowering phase [11,12,13,14,15,16,17,18]. To date, adventitious shoot regeneration systems and *A. tumefaciens*-mediated transformation methods of leaf explants have been reported for several blueberry cultivars. Some blueberry transgenic lines have shown the ability to survive low temperatures or to flower precociously or persistently [13,17]. High concentrations of common cytokines zeatin (ZT) and 6-(γ,γ-Dimethylallylamino) purine (2-ip) can induce shoot regeneration from leaf explants of *cv.* ‘Aurora’, ‘Brigitta’, ‘Elliott’, ‘Bluecrop’, and ‘Legacy’ [19]. In addition, thidiazuron (TDZ) and a-naphthaleneacetic (NAA) are commonly used to induce adventitious bud regeneration from blueberry leaves [20]. The shoot regeneration medium supplemented with modified WPM (reported by Rowland and Ogden in 1992), 1.0 mg·L^−1^ TDZ, and 0.5 mg·L^−1^ NAA was regarded as the best combination for leaf explants of most cultivars of highbush blueberry [19]. The combination of ZT and IBA has been demonstrated to effectively induce buds from callus derived from certain blueberry cultivars, particularly *cv.* ‘Red Button’, with the highest rates of callus induction and browning being 90.3% and 33.3%, respectively, which are influenced by genotype [21]. This phenomenon has also been observed in other research that used different types or concentrations of plant growth regulators [19,20]. Moreover, 83.33% of callus showed green, fluorescent signals, while only 12.82% efficiency was achieved in obtaining transgenic buds from the induced callus [21]. Despite the combination of different plant growth regulators for shoot regeneration systems and subsequent transformation techniques, such as *A. tumefaciens*-mediated transformation methods, being carried out in several blueberry cultivars, all these transformations involve inducing transgenic buds from leaf discs, which is time-consuming and requires several years for plant cultivation before conducting research on fruit quality and flowering [17,19,21]. Furthermore, these methods have not yet proven to be highly efficient approaches to transform genes of interest, as reported transgenic plant acquisition rates range between 5–12.82%, influenced by factors such as genotype, co-cultivation time, *A. tumefaciens* strain and concentration, explant age, and regeneration system [19,21,22]. Additionally, blueberries, being woody plants, require a long period to stably bear fruit, which limits further research on molecular mechanisms of fruit development, fruit ripening, anthocyanin accumulation, abiotic stress, and other basic research.

The callus induction and transformation system has been widely applied to fruit trees such as citrus, apple, chestnut, pear, etc., due to the advantages of time saving, fast growth, and high efficiency [23,24,25,26]. Common explants used for callus induction include mature seeds, immature embryos, leaves, stem segments, anther, etc. [27,28,29,30]. Frequently, plant growth regulators, such as 2, 4-D, 6-BA, TDZ, NAA, etc., are added to induction mediums [31,32,33]. At present, the mechanism of *VcMYB4a* in abiotic stress verified in blueberry callus only refers to apple callus transformation methods [16]. However, the callus regeneration and transformation system has not been reported in blueberries. Therefore, it is necessary to establish a highly efficient induction and transformation system for blueberry callus.

Anthocyanin is an important secondary metabolite in the flavonoid family which can attract pollinators and protect plants from visible light and UV-B radiation [34,35]. It is abundant in blueberries, which have antioxidant activities and protective effects on the cardiovascular system. The chalcone synthetase (*CHS*) gene provides a prerequisite for anthocyanin synthesis and is an indispensable substance in the synthesis process [36]. Moreover, chalcone is upstream in the flavonoid synthesis pathway, which can be induced by UV-B and low temperature, and the catalytic reaction of chalcone synthetase is an important rate-limiting step of the flavonoid synthesis pathway [34,37]. *CHS* is a key enzyme gene in the anthocyanin biosynthesis pathway. It has been reported that high light (HL) stress, causing red coloration of leaves, can enhance *CHS* gene expression levels in *CHS*-overexpression lines to improve plant adaptability to HL [38].

In this study, we established a stable callus regeneration system for leaf explants of blueberry. Then, we also concentrated on several crucial factors, including hygromycin sensitivity, co-cultivation time, and *Agrobacterium* concentration, that have been reported to significantly influence *A. tumefaciens*-mediated transformation efficiency. Finally, we summarized a highly efficient transformation method for leaf callus of blueberry. The new method for blueberries is reliable, improves the transformation efficiency by up to 49.02%, and supports for gene functional research and molecular breeding.

## 2. Results

### 2.1. Leaf Callus Induction and Proliferation of Blueberry cv. ‘Legacy’

In order to find out the suitable callus induction medium for leaf explants of *cv*. ‘Legacy’, the medium was added with MS or WPM separately, containing different concentrations of 2, 4-D (Figure S1 and Table S1). The leaves on MS medium produce the green and close callus, while the leaves on WPM medium induce the yellow and soft callus. To obtain more callus, leaves were cultivated on WPM medium with different concentrations of 2, 4-D. We found that 1 or 2 mg·L^−1^ 2, 4-D efficiently induced a more yellow and soft callus. But callus in this combination grew slowly, and only a little callus was obtained for two months. When the concentration of 2, 4-D increased to 4 mg·L^−1^, callus growth was limited, and the color became brown and hard to induce new callus.

**Figure 1:**
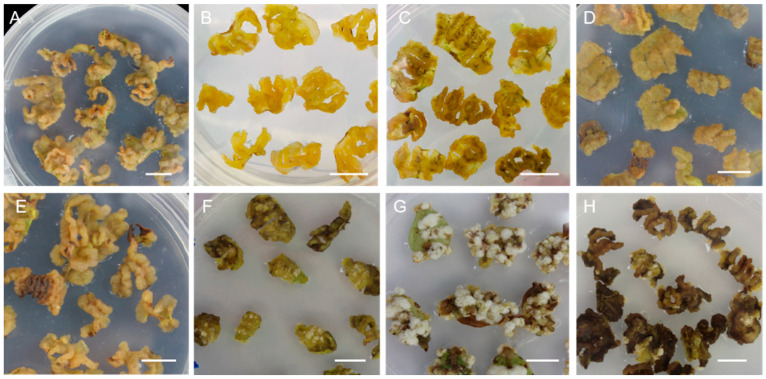
Leaf growth status of *cv.* ‘Legacy’ in callus induction medium containing different combinations of 2,4-D and 6-BA. (**A**–**D**) Leaf growth status in WPM containing 1 mg·L^−1^ 2,4-D and different concentrations of 6-BA (0.2, 0.4, 0.6, 0.8 mg·L^−1^), respectively. (**E**–**H**) Leaf growth status in WPM containing 4 mg·L^−1^ 2,4-D and different concentrations of 6-BA (0.2, 0.4, 0.6, 0.8 mg·L^−1^), respectively. The scale in the figure is 1 cm.

Therefore, we added different concentrations of 6-BA (0.2, 0.4, 0.6, 0.8 mg·L^−1^) to the WPM medium containing different concentrations of 2, 4-D (1 or 4 mg·L^−1^) to obtain more healthy, yellow, soft callus from leaf explants (Figure 1A–H and Table S2). We found that leaf explants induced different conditions of callus in different combinations significantly after 30 days. When the WPM medium was supplemented with 1 mg·L^−1^ 2, 4-D and 0.2 mg·L^−1^ 6-BA, the callus was compact and a grayish yellow (Figure 1A). When the WPM medium supplemented with 1 mg·L^−1^ 2, 4-D and 0.4 or 0.6 mg·L^−1^ 6-BA, the callus was yellow, soft, loose, and grew rapidly, as we harvested callus after 30 days (Figure 1B,C). When the concentration of 2, 4-D increased to 4 mg·L^−1^ with the supplement of 6-BA, the callus became brown and grew some white tissue (Figure 1E–H). Therefore, the WPM medium added with 1 mg·L^−1^ 2, 4-D and 0.4 mg·L^−1^ 6-BA was the optimum callus induction medium for *cv.* ‘Legacy’ and was suitable for proliferating new callus in the darkness at 25 °C for 30 days (Figure 1B).

Since there was no significant difference in inducing callus proliferate between 1 and 2 mg·L^−1^ for the concentration of 2, 4-D, we took the average, 1.5 mg·L^−1^, as the suitable concentration for callus proliferation medium (Figure S2A,B). The callus proliferation medium was supplemented with 1.5 mg·L^−1^ 2, 4-D and different concentrations of 6-BA (0.2, 0.4, 0.6, 0.8 mg·L^−1^) (Table S3). When the concentration of 6-BA in the subculture medium increased to 0.4 mg·L^−1^, the callus not only became yellow, soft, and loose, but also grew quickly, and we obtained a mass of loose and healthy callus after it was sub-cultured for 30 d. Therefore, the optimum proliferation medium for callus of *cv.* ‘Legacy’ was WPM medium supplemented with 1.5 mg·L^−1^ 2, 4-D and 0.4 mg·L^−1^ 6-BA in the darkness at 25 °C every 30 days (Figure S3).

### 2.2. Leaf Callus Induction and Proliferation of Blueberry cv. ‘Northland’

**Figure 2:**
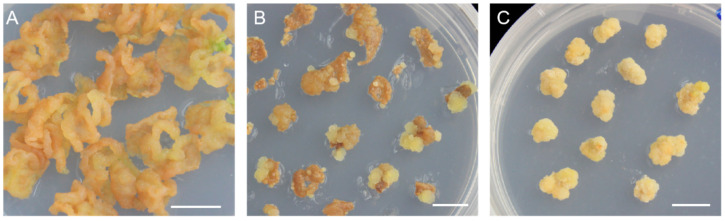
The callus growth process of *cv.* ‘Northland’. (**A**) Leaves of *cv.* ‘Northland’ were cultured in WPM + 1 mg^−1^ 2,4-D + 0.4 mg^−1^ 6-BA medium for 40 d. (**B**) The callus of *cv.* ‘Northland’ was removed from the leaves and cultured in WPM + 1.5 mg^−1^ 2,4-D + 0.4 mg^−1^ 6-BA for 20–30 d to produce yellowish spherical callus and grow rapidly. (**C**) The callus of *cv.* ‘Northland’ was cultivated in WPM + 1.5 mg^−1^ 2,4-D + 0.4 mg^−1^ 6-BA medium for 30 days. The scale in the figure is 1 cm.

In order to further verify whether this system is suitable for other cultivars, we conducted a verification in *cv.* ‘Northland’. The leaf explants of *cv.* ‘Northland’ were cultivated in WPM + 1 mg·L^−1^ 2,4-D + 0.4 mg·L^−1^ 6-BA and induced yellow, soft callus (Figure 2A). After 40 days, the leaves of *cv.* ‘Northland’ which had induced callus were cut into small pieces, transferred in proliferation medium, and then produced new callus for 20~30 d (Figure 2B). Then, the callus was taken out from the small pieces of leaves and transferred to new proliferation medium and proliferated quickly (Figure 2C). The results indicated that the induction medium and proliferation medium selected in this study were also suitable for *cv.* ‘Northland’.

### 2.3. Selection of Cefotaxime and Hygromycin Concentration

**Figure 3:**
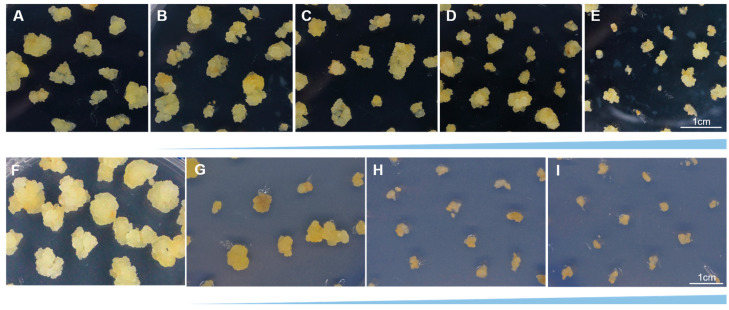
Callus of *cv.* ‘Northland’ growth status in different concentrations of cefotaxime and hygromycin. (**A**) CK. (**B**–**E**) Callus growth status in different concentrations of Cef (100, 200, 300, 400 mg·L^−1^). (**F**) CK. (**G**–**I**) Callus growth status in different concentrations of Hyg (30, 50, 80 mg·L^−1^). The scale in (**A**–**D**) is the same as in (**E**) and the scale in (**F**–**H**) is the same as in (**I**).

The bacteriostatic agent type and concentration significantly inhibited growth of explants, which was necessary to screen optimum concentration of bacteriostatic agent to reduce its influence on plant tissue growth. The Cef was chosen as the bacteriostatic agent to limit the growth of *A. tumefaciens*, and Hyg was used to identify the transgenic callus. In order to select the optimum cefotaxime and hygromycin concentration, callus was transferred to the selection medium containing different concentrations of Cef and Hyg. The callus grew rapidly when it was cultivated in the control group (CK) without Cef and Hyg (Figure 3).

Here, we found that when the concentration of Cef was between 100 to 200 mg·L^−1^, callus growth was limited (Figure 3B,C). Until the concentration of Cef was between 300 to 400 mg·L^−1^, the callus could not grow up obviously (Figure 3D,E). In order to reduce the inhibition of callus growth, we chose 200 mg·L^−1^ as the optimum concentration of Cef, since the concentrations of 100~400 mg·L^−1^ inhibited bacteria growth effectively (Figure 3C).

When the concentration of Hyg was 30 mg·L^−1^, callus growth was limited and became yellowish white and loose. When the concentration of Hyg increased to 50 mg·L^−1^, callus growth was limited obviously, and it gradually browned and died. Therefore, we chose 50 mg·L^−1^ Hyg as the optimum concentration of Hyg to select for the Hyg-resistant callus (Figure 3H).

### 2.4. Agrobacterium-Mediated Transformation of Callus

In order to establish an *Agrobacterium*-mediated stable transformation for *cv.* ‘Northland’, we used EHA105 containing the binary vector pMDC85 to infect the callus, and nine different treatment schemes were designed for bacteria liquid concentration (value of OD_600_) infection time and co-cultivation time through the L_9_ (3^3^) orthogonal table to select the best combination for the transformation system (Table 1).

The highest rate of GFP signal detected in the transformed callus was 49.02%, which was observed at OD_600_ = 0.7 after 10 min infection and 4 days’ co-cultivation (Table 1). According to the value of quadratic sum and significance (*p* < 0.05), the influence of different factors on the genetic transformation efficiency of the callus of *cv.* ‘Northland’ were sorted by size as infection time > bacteria liquid concentration > co-cultivation time through variance analysis. Infection time influenced transformation efficiency significantly, as bacteria liquid concentration and co-cultivation time had no significant effect, which indicated that infection time is the main influencing factor for transformation efficiency (Table 2). Compared with the mean value, the longer the infection time, the lower the efficiency of callus transformation. When the infection time increased to 30 min, the transformation efficiency was 7.15%, which was the lowest of all treatments (Table 3). Therefore, the optimum transformation treatment was observed at OD_600_ = 0.7 for bacteria liquid concentration after 10 min infection and 4 days’ co-cultivation, according to the mean value and the rate of GFP signal detected in the transformed callus.

### 2.5. Molecular Identification and GFP Detection for Transgenic Callus

**Figure 4:**
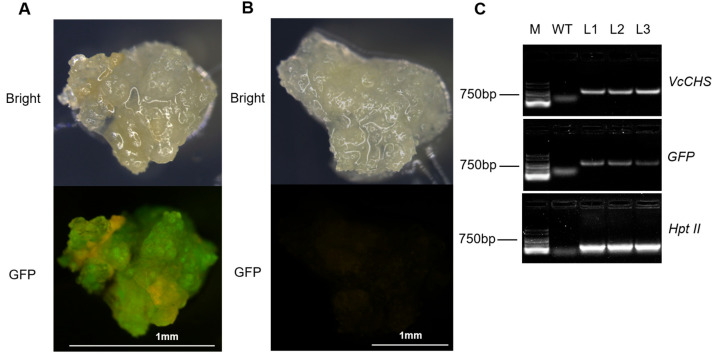
Identification of the GFP signal and PCR detection. (**A**) Green fluorescence was observed in the transformed callus. (**B**) Green fluorescence was not observed in the wild-type callus. (**C**) PCR detection of the *VcCHS*, *GFP*, and *Hpt II* gene, non-transgenic sample (WT), and 3 transgenic lines (L1–L3).

**Figure 5:**
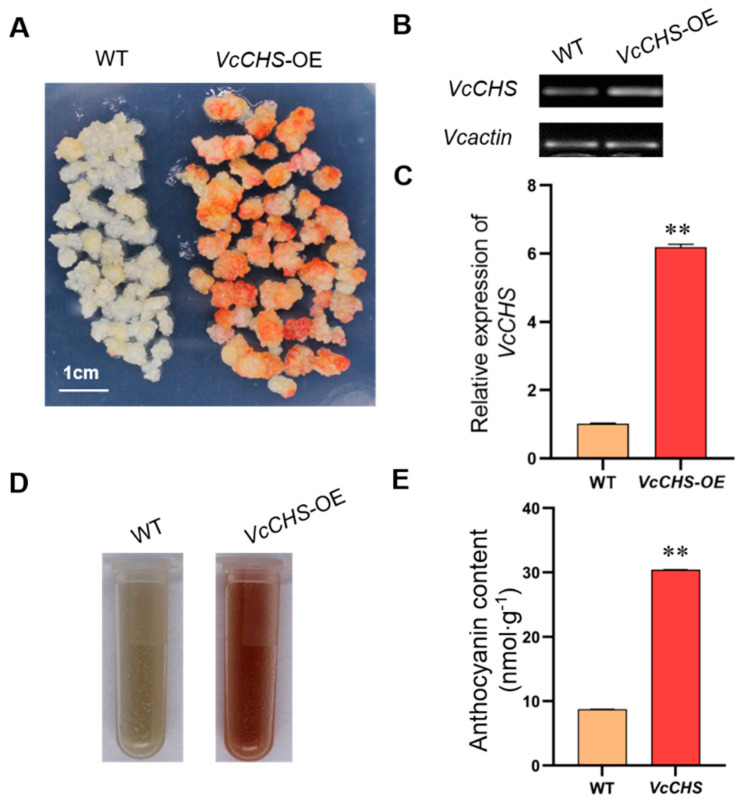
Phenotype analysis of the wild-type callus and *VcCHS* transgenic callus and RT-qPCR detection. (**A**) Red callus was observed in the transformed callus, while the WT callus showed only a small amount of dot coloring. (**B**) Analysis of *VcCHS* transcriptional levels in WT callus and transgenic lines by RT-PCR analysis. (**C**) Relative expression level of *VcCHS* gene in WT and transgenic callus (*VcCHS*−OE). ** indicated extremely significant difference. (**D**) Anthocyanin content of WT and *VcCHS*−OE. (**E**) Analysis of anthocyanin content of WT and *VcCHS*−OE. ** indicated extremely significant difference.

The wild-type callus (WT, non-transformed callus) and transgenic callus was identified by stereo fluorescence microscope. Obvious green fluorescence was observed in the transgenic callus, while the WT showed no green fluorescence (Figure 4A,B). In order to further identify the resistant callus by molecular techniques, callus containing GFP signal was selected to detect exogenous *VcCHS* and gene insertion by PCR (Figure 4C). Clear bands could be observed in the transgenic callus. The semi-quantitative detection of both callus showed that no bands were observed in the WT callus, while the expression level of *VcCHS* genes in the transgenic callus was 6 times that of the WT callus (Figure 5B,C).

### 2.6. Phenotype Analysis of Transgenic Callus

The WT callus and *VcCHS* transgenic callus grown for 20 days was transferred in continuous low temperatures containing white and UV light. The transgenic callus exposed to light showed a large area of coloring and red on day 5, while the WT callus showed only a small amount of dot coloring (Figure 5A,D). The analysis of anthocyanin content showed that there was a significant difference in anthocyanin content between the WT callus and overexpressed *VcCHS* transgenic callus, indicating that overexpressed *VcCHS* transgenic callus could significantly increase anthocyanin accumulation in callus of *cv*. ‘Northland’ (Figure 5E).

## 3. Discussion

Callus induction efficiency was significantly correlated with plant genotype, combination and concentration of plant growth regulators, light intensity, and culture medium [39,40,41,42]. In other plants, many studies have shown that different genotypes of the same species have significant effects on callus induction efficiency, and that even different parts of the same genotype plant have different induction efficiency [43]. In this study, the callus induction system constructed by the *cv.* ‘Legacy’, including culture medium, plant growth regulators, and culture condition, was found to be suitable for *cv.* ‘Northland’, indicating that these two plant growth regulators may have a certain universality in inducing callus of northern highbush blueberry. The leaf explants were also wounded before transformation because it was easier to induce the callus after injury [44]. In addition, reports of callus induction in other plants also showed that 2, 4-D could induce callus from different types of explants, and 6-BA had a significant effect on accelerating callus generation and propagation [45]. This experiment also verified the research that the culture medium we obtained had better effects on callus induction for two blueberry cultivars. Some plants had reported that the suitable culture medium for one genotype does not work well for others [31,46]. Therefore, whether this callus induction system is suitable for other genotypes needs to be proved by further experiments.

It was reported that the *Agrobacterium* strain and concentration, genotype, explant type, and co-cultivation time significantly influence transformation efficiency [47,48,49,50]. There are different kinds of antibiotics to distinguish transgenic material from explants, such as kanamycin (Km) and hygromycin (Hyg), which were frequently applied to *A. tumefaciens*-mediated transformation methods for some woody plants [51,52]. The tolerance concentration of wild-type materials adapted to antibiotics was the key factor to screen, since the growth of wild-type plant materials was inhibited at a certain critical concentration [53]. Therefore, screening antibiotics could effectively identify transformed plant materials and improve the screening efficiency of positive transgenic materials. The infection time and co-cultivation were also key factors. If the infection time was short, *A. tumefaciens* could not fully contact plant materials; if the infection time was too long, the growth of plant materials was inhibited or even caused explants to die. If the culture time was too short, the infection effect of *A. tumefaciens* was affected and the positive rate of the material was reduced; on the contrary, if the culture time was too long, the overgrowth of *A. tumefaciens* would pollute the growth of plant materials, which was difficult to clean and even led to the death of plant materials [54]. In this study, Hyg was chosen as the screening antibiotic and Cef as a bacteriostatic agent. The tolerance of *cv.* ‘Northland’ callus was assessed, and the concentration at which callus growth was significantly inhibited was selected as the screening concentration. When determining the concentration of Cef for screening, we aimed to select a treatment with low concentration and strong bacteriostatic effect, in order to minimize its impact on callus proliferation. Some studies have suggested that yellow callus is more suitable for genetic transformation [55]. In line with this, we obtained light yellow callus that exhibited loose structure and rapid proliferation, similar to other species used for genetic transformation. The transformation efficiency reached 49.02%, indicating significant improvement in the *A. tumefaciens*-mediated transformation method developed for *cv.* ‘Northland’ callus. Furthermore, the rate of GFP signal detected in transformed callus correlated with PCR detection results, confirming stability, reliability, and high efficiency of the transformation system in this study. Transgenic callus was successfully generated, and RT-qPCR analysis revealed a significant six-fold increase in *VcCHS* expression compared to wild-type.

There is no doubt that it is valuable for the transformation system of plants to obtain regenerated plantlets. However, it is also worth noting that direct plantlet generation from callus can be challenging, especially for fruit trees and woody plants, because of the high heterozygosity. On the other hand, this process requires specific hormones, conditions, and a lot of time to cultivate the transgenic plants to harvest. Compared to the transgenic seedlings that require a long period of time, *A. tumefaciens*-mediated callus transformation could be rapidly and directly carried out for gene function verification and phenotype study. For example, callus transformation system is commonly utilized in coloration assays to reveal the molecular mechanisms underlying anthocyanin biosynthesis, abiotic stresses, and fruit quality in apples and grapevines [56,57,58,59,60,61,62,63]. Therefore, it had unique advantages and accelerated the research on agronomic characteristics of woody plants or fruit trees. In addition, it can also be used for the synthesis of bioactive secondary metabolites through transgenic callus instead of generating transgenic plants [64]. It has been reported that high added-value plant compounds were obtained through transgenic callus [65,66]. This will provide a reference for mass synthesis of bioactive secondary metabolites that have medicinal and health values in the future.

## 4. Materials and Methods

### 4.1. Plant Materials and Callus Induction

Stem segments of highbush blueberry *cv.* ‘Legacy’ and ‘Northland’ were cultured at 25 °C for 30 days in order to collect the explants to obtain tissue culture seedlings. Leaf explants cultivated on the shoot stock culture medium for 45–50 d were used. To select the suitable callus induction medium for leaf explants, leaves of *cv.* ‘Legacy’ were wounded transversely with a scalpel and placed on woody plant medium (WPM) supplemented with 30 g·L^−1^ sucrose, 6.4 g·L^−1^ agar, and different concentrations of 2,4-D (1, 2, 4 mg·L^−1^) and 6-BA (0.2, 0.4, 0.6, 0.8 mg·L^−1^); then, the explants were cultured at 25 °C in the darkness. Callus growth condition and color were recorded after 35 days. The callus was transferred onto the callus proliferation medium supplemented with WPM, 30 g·L^−1^ sucrose,6.4 g·L^−1^ agar, 1.5 mg·L^−1^ 2,4-D, and different concentrations of 6-BA (0.2, 0.4, 0.6, 0.8 mg·L^−1^), and cultured at 25 °C in the darkness. The callus growth condition and color were recorded after 35 days. The pH of all the medium was kept 5.2~5.5 before adding agar and autoclaving at 121 °C for 20 min. The callus was sub-cultured on the fresh proliferation medium every 4 weeks. After two subcultures in the darkness, the callus which were yellow and loose were chosen for the transformation experiment.

### 4.2. Selection of Antibiotics Concentration

In order to screen the transgenic callus and inhibit the growth of *A. tumefaciens*, wild-type callus (WT, non-transformed callus) of *cv.* ‘Northland’ was cultivated in selection medium containing different concentrations of Hyg (0, 30, 50, 80 mg·L^−1^) and Cef (0, 100, 200, 300, 400 mg·L^−1^) to observe the tolerance value in the darkness at 25 °C. Fifty callus clumps was used for each treatment and repeated 3 times. The callus growth condition and survival rate were recorded after 30 days.

### 4.3. Plasmid Construction

The binary vector pMDC85 with kanamycin resistance gene, hygromycin resistance gene, and green fluorescent protein gene (*GFP*) was used in this study. The genomic fragment of *VcCHS* was cloned with gene-cloning primers through blueberry genomic DNA. The PCR products for *VcCHS* were constructed into the binary vector pMDC85, and the promoter was 35S, while the expression vectors pMDC85-*VcCHS* were transformed into *A. tumefaciens* strain EHA105.

### 4.4. Agrobacterium-Mediated Transformation for Blueberry Callus

The *A. tumefaciens* strain EHA105 was cultivated overnight in YEB liquid medium supplemented with 50 mg·L^−1^ rifampicin and 50 mg·L^−1^ kanamycin at 120 rpm at 28 °C, until OD_600_ was 0.8~1.0. Bacteria liquid was transferred into a 50 mL tube to centrifuge at 5000 rpm for 10 min and then resuspended in WPM liquid medium with 20 g·L^−1^ sucrose and 100μM Acetosyringone (AS). The callus was transferred into different bacteria liquid concentrations (OD_600_ = 0.4, 0.7, 1.0) and infected with *A. tumefaciens* for 10, 20, or 30 min in the darkness, then operated in shake cultivation in 120 rpm at 28 °C. After being dried through filter paper, callus was co-cultivated on regeneration medium extra containing 100 μM AS with *A. tumefaciens* for 2, 3, or 4 days in the darkness. Then, callus was transferred onto the selection medium that regeneration medium supplemented with 50 mg·L^−1^ hygromycin and 200 mg·L^−1^ cefotaxime (Cef), and the selection medium were updated every 20 days. After 50 days, the resistant callus produced a new callus and continued cultivating on the selection medium, and the unsuccessfully infected callus became brown and stopped growing.

### 4.5. Molecular Identification of Transgenic Callus

The transgenic callus was identified by stereo fluorescence microscope with a GFP filter and observed in 450~490 nm.

The total genomic DNA of transgenic callus was isolated by the method of CTAB. The DNA of WT and binary vector pMDC85 were the control group for polymerase chain reaction (PCR). The primers corresponding to a 963 bp fragment of the coding region of *VcCHS* were 5′-ATGGGAATCATTCCAGAGTCTCCTCT-3′ and 5′-CTAGATCTCCCCATCCCAGAGCTCCT-3′. The PCR reaction conditions were 94 °C for 3 min, 30 cycles of 98 °C for 10 s, 60 °C for 20 s, and 72 °C for 70 s, and then 72 °C for 5 min. The products of PCR were detected in 1% agarose gel. The qRT-PCR was carried out in ABI-StepOnePlu system, the reaction conditions were followed as PowerUp^TM^ AYBRTM Green Master Mix in ABI. The relative transcriptional expression levels of each gene were calculated by 2^−ΔΔCt^. The primers of *VcCHS*-forward were 5′-TCAACCAACGCAACGATTCCAG-3′ and *VcCHS*-reverse were 5′-ACAATGCTCCGACTGGTAAACG-3′. The primers of *VcActin*-forward were 5′-ACACGGGGAGGTGTGACAA-3′ and *VcActin*-reverse were 5′-CCTCCAATGGATCCTCGTTA-3′.

### 4.6. Anthocyanin Content Detection

The positive transgenic callus and WT callus was cultivated in a manual climatic box at 14 °C under continuous white light (light intensity of 10,000 lx) containing UV-B. After 5 days, the color of callus was recorded, and the anthocyanin of callus was extracted by using 1% (*v*/*v*) HCl-alcohol. The procedures were as follows: 0.5 g callus was ground into powder with liquid nitrogen and transferred into a 2 mL tube, adding anthocyanin extract liquid (95% alcohol + 1.5 mol·L^−1^ HCl) at 4 °C for 24 h in the darkness. The samples were centrifuged at 5000 rpm for 5 min and obtained supernatant, detected to have a spectrophotometric quantification of 530, 620, and 650 nm. Anthocyanin content = OD _λ_/ξ _λ_ × V/M × 10^6^, OD _λ_(anthocyanin density) = (A_530_ − A_620_) − 0.1×(A_650_ − A_620_), ξ _λ_(extinction coefficient of anthocyanin at 530 nm) = 4.62 × 10^4^, V = extraction volume, M = callus weight. The experiments were repeated three times independently [60].

### 4.7. Statistical Analysis

Data for callus induction and stable transformation were analyzed for significance by analysis of Duncan’s test in ANOVA through SPSS 19.0. The figures were made by SigamPlot 2018.

Callus induction efficiency = the number of leaf explants generates callus/Total leaf explants per dish ×100%. The rate of GFP signal detected in the transformed callus (%) = the number of callus containing GFP signal/The number of transformed callus ×100%.

## Figures and Tables

**Table 1:** Orthogonal experimental results of L_9_(3^3^) for the transformation of *cv.* ‘Northland’ callus in different conditions.

Influence Factors			Rate of Resistance (%)	Rate of GFP Signal (%)
OD_600_	Infection Time (min)	Co-Cultivation Time (day)		
0.4	10	2	34.29	25.71
0.4	20	4	11.11	11.11
0.4	30	3	10.20	8.16
0.7	10	4	49.02	49.02
0.7	20	3	23.08	23.08
0.7	30	2	6.52	6.52
1.0	10	3	27.27	27.27
1.0	20	2	7.55	3.77
1.0	30	4	6.78	6.78

**Table 2:** Variance analysis of factors affecting the efficiency of genetic transformation in *cv.* ‘Northland’ callus.

Source of Variation	Quadratic Sum	Degree of Freedom	Mean Square	F	*p*
Error correction model	1693.367 ^a^	6	282.228	12.688	0.075
Intercept	2895.157	1	2895.157	130.153	0.008
A: OD_600_	316.395	2	158.198	7.112	0.123
B: Infection time	1206.674	2	603.337	27.123	0.036 *
C: Co-cultivation time	170.299	2	85.149	3.828	0.207
Error	44.488	2	22.244		
Summation	4633.013	8			

Note: * indicates a significant difference (*p* < 0.05). ^a^ represented R square =0.974 (adjusted R square =0.898).

**Table 3:** ANOVA analysis of infection time influence on transformation frequency for *cv.* ‘Northland’ callus.

	Infection Time (min)			F	*p*
	10	20	30		
Transformation efficiency (%)	34 ± 13.03	12.65 ± 9.75	7.15 ± 0.88	6.815	0.029 *

Note: * indicates a significant difference (*p* < 0.05). The data was the mean value ± SD (*n* = 3).

## Data Availability

All relevant data can be found within the manuscript and its Supplementary material.

## Supplementary Materials

The following supporting information can be downloaded at: https://www.mdpi.com/article/10.3390/plants12162905/s1, Figure S1: Leaf growth status of *cv.* ‘Legacy’ in WPM or MS containing different combinations of 2,4-D; Figure S2: Callus growth status of *cv.* ‘Legacy’ in WPM containing different combinations of 2,4-D; Figure S3: The growth status of callus of *cv.* ‘Legacy’ in WPM + 1.5 mg^−1^ 2,4-D + 0.4 mg^−1^ 6-BA medium for 30 days; Table S1: Effects of different mediums and 2,4-D concentrations on callus induction for *cv.* ‘Legacy’; Table S2: Effects of different plant growth regulator combinations on callus induction for leaves of *cv.* ‘Legacy’; Table S3: Effects of different concentrations of 6-BA on the subculture of callus of *cv.* ‘Legacy’.
