# Supplementary material for: An Efficient Transformation System for Fast Production of VcCHS Transgenic Blueberry Callus and Its Expressional Analysis
Source: Plants (Basel). 2023 Aug 9;12(16):2905. doi: 10.3390/plants12162905 (PMC10458251; doi:10.3390/plants12162905)
Supplement: Supplementary file 1 [file plants-12-02905-s001.zip › plants-2516401-supplementary.pdf]

Supplementary File

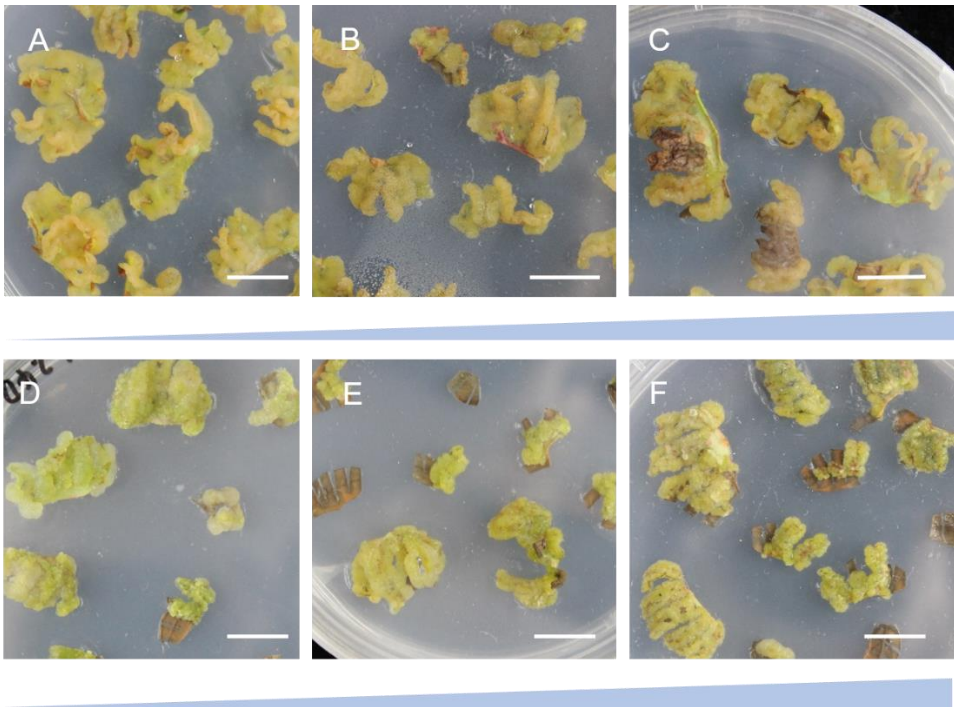

Figure S1 Leaves growth status of *cv.* 'Legacy' in WPM or MS containing different concentrations of 2,4-D

A-C: WPM containing different concentrations of 2,4-D (1, 2, 4 mg·L^{-1}); D-F: MS containing different concentrations of 2,4-D (1, 2, 4 mg·L^{-1}). The scale in the figure is 1cm.

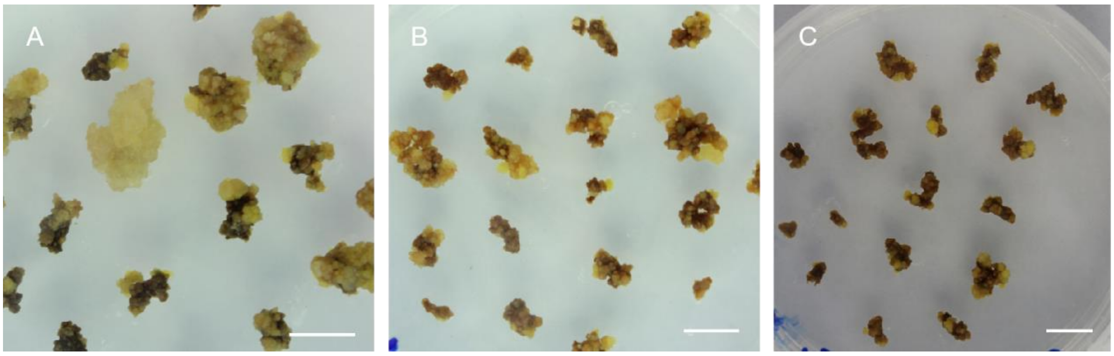

Figure S2 Callus growth status of *cv.* 'Legacy' in WPM containing different concentrations of 2,4-D

A-C: WPM containing different combinations of 2,4-D (1, 2, 4 mg·L^{-1}). The scale in the figure is 1cm.

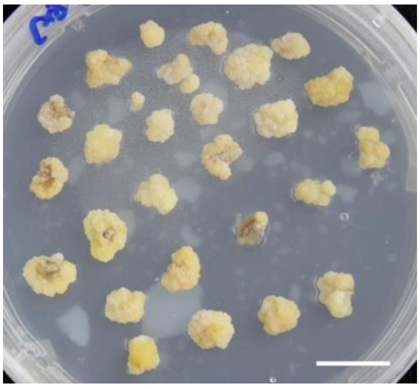

Figure S3 The growth status of callus of *cv.* ‘Legacy’ in WPM+1.5 mg·L^{-1} 2,4-D+0.4 mg·L^{-1} 6-BA medium for 30 days. The scale in the figure is 1cm.

Table S1 Effects of different combinations of medium and 2,4-D on callus induction for *cv.* ‘Legacy’

**Medium**	**2,4-D（mg·L^{-1}）**	**Cultivation time （d）**	**Callus induction condition**
MS	1	35	Leaf curl, leaf surface grow thin light green callus
	2	35	
	4	35	
WPM	1	35	Leaf curl, surface and wound developed orange callus
	2	35	
	4	35	

Table S2 Effects of different combinations of plant growth regulator on callus induction for leaves of *cv.* ‘Legacy’

**Medium**	**2, 4-D （mg·L^{-1}）**	**6-BA （mg·L^{-1}）**	**Cultivation time（d）**	**Growth status**
WPM	1	0.2	35	Orange callus developed from the leaf wounds
	1	0.4	35	Light yellow callus developed on the leaf surface
	1	0.6	35	Yellow callus developed on leaf surface
	1	0.8	35	Dark yellow callus developed from leaf wounds
	4	0.2	35	There was no obvious callus

			inducing from the leaves, and white particles were formed at the wound
4	0.4	35	
4	0.6	35	
4	0.8	35	

Table S3 Effects of different concentrations of 6-BA on the subculture of callus of *cv*. ‘Legacy’

**Medium**	**2,4-D（mg·L^{-1}）**	**6-BA（mg·L^{-1}）**	**Cultivation time（d）**	**Callus growth condition**
WPM	1.5	0.2	30	Pale yellow and grows slowly
	1.5	0.4	30	Light yellow and grows rapidly
	1.5	0.6	30	light yellow and grows rapidly
	1.5	0.8	30	Pale yellow and grows slowly
